# For Yeast Protein Hubs, More Data Means More Connections

**DOI:** 10.1371/journal.pbio.0040331

**Published:** 2006-09-19

**Authors:** Richard Robinson

What happens inside a cell? To a good first approximation, the answer is “thousands of proteins interact.” A cell’s form, and all its functions, arises from those interactions. One goal of systems biology is to describe those interactions—by focusing not just on this organelle or that signaling pathway, but on the entire network of proteins within the cell—and then to deduce the patterns of interactions that control that network.

Initial analysis of such protein networks in the budding yeast *Saccharomyces cerevisiae* has led to a hub-centric view of interactions, in which a small number of proteins, the hubs, interact with a disproportionately large number of other proteins. In this model, the hubs form the basis for functional “modules” that perform discrete tasks in the cell. Such modules have been thought to be physically and functionally discrete from other modules, so that there is much interaction within the module, and relatively little between modules. In particular, it has been proposed, hubs of different modules tend not to interact with one another. One version of this model further suggests two types of hubs: “party” hubs are co-expressed and co-localized with most members of their module (together creating a party), while “date” hubs are not, instead engaging in a series of temporally and/or spatially distinct interactions (dates), including interactions with partners in other modules.

Models are only as good as the information they are based on, though. A new study by Nizar Batada, Laurence Hurst, Mike Tyers, and colleagues, combining data from several large budding yeast data sets, shows a much higher degree of interaction between hubs from different clusters, and finds no evidence for the date–party distinction. Thus, the global network appears more homogeneous and perhaps harder to tease into discrete modules than anticipated.

The authors began by integrating data from multiple large data sets of yeast protein interactions, to create a unified set of over 9,000 interactions among almost 3,000 proteins, more than three times as much information on which previous models have been built. As in previous work, certain proteins—the hubs—emerge as being highly connected, binding to and interacting with many more partners than expected by chance. But the previously identified trend for hubs to avoid interaction with one another disappeared in the large data set, a result that persisted as the data were sifted through several different kinds of analytic filters. Hubs are still real and, according to this result, frequently are among the many proteins they interact with.

**Figure:**
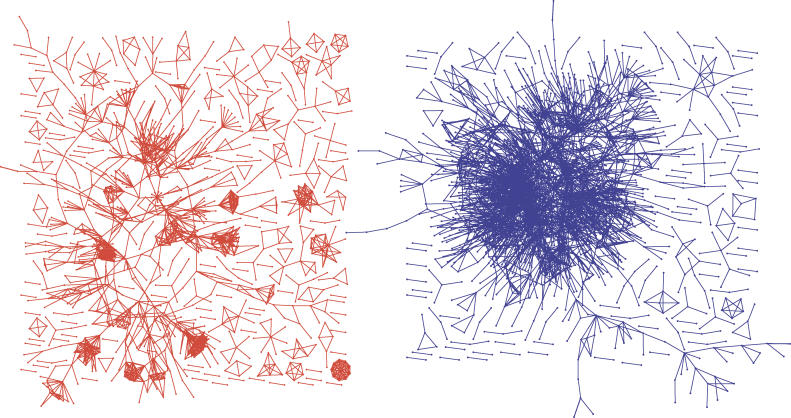
Global organization of yeast interaction networks. The smaller filtered yeast interactome (FYI) network (left) contains locally dense regions that are sparsely interconnected, whereas the larger high-confidence FYI (HC^fyi^) network (right) is densely interconnected overall, suggestive of extensive coordination and dependencies among diverse processes.

Because of the proposed role of date hubs as vital linkages between modules, deletion of date hubs should cause a collapse of the entire network. This result was indeed seen in smaller data sets, and gave support to the initial concept of date hubs. In the larger set, however, no such collapse occurs, as many alternative links still exist between modules even after the deletion of putative date hubs. Neither does the date-versus-party distinction emerge from analysis of co-expression: some hub proteins are co-expressed with their interacting partners more than others, but there is a continuous range from massive co-expression to very little co-expression, not two distinct classes of co-expression behavior. Finally, it has been predicted that date hubs evolve more quickly than party hubs, because their intermodular function allows them more flexibility than a party hub, whose function (it has been argued) is more rigidly fixed by its role within its module. When the authors tested this prediction for yeast protein evolution, no such correlation emerged. Together, these data indicate that the date–party distinction is more likely a property of the small data set it was developed from than a bona fide attribute of the global yeast protein interaction network.

A key finding of this study is that there is a generally higher level of connectivity between clusters that were once thought to be relatively isolated functionally. The authors liken the structure of the earlier model to altocumulus clouds: dense, billowy clouds connected by the thinnest of wisps. A more appropriate analogy, they say, might be stratus clouds: a thick cloud cover with lumps and thinner spots, not uniform but not discrete either. One consequence of this connectivity structure is that functional modules may be harder to physically delineate than has been previously thought, at least in yeast (modules do appear to be more discrete in prokaryotes). Another consequence is that hub–hub interactions, which often reflect essential connections, may form the critical regulatory backbone of the cell.

One alluring feature of the previous altocumulus model of connectivity was that, with few hub–hub interactions, the problem of inadvertent activation of a module by a distant hub was minimized. Instead, the stratus model suggests that such cross-talk may be an important problem for the cell, and that tight control of hub–hub interactions is likely to be a feature of hub regulation; initial evidence suggests this is indeed the case. Further exploration of these and other predictions may clarify the usefulness of the stratus model in developing a systems-level understanding of the cell.

